# Comparative Pathogenicity of PCV2, PCV3, and PCV4 in Piglets: Insights Into Clinical, Pathological, and Immunological Features

**DOI:** 10.1155/tbed/6362100

**Published:** 2025-07-09

**Authors:** Jiawei Zheng, Xue Li, Xinru Lv, Yaqi Han, Xinwei Zhang, Si Chen, Fuxian Zhang, Linzhu Ren

**Affiliations:** ^1^College of Animal Sciences, State Key Laboratory for Diagnosis and Treatment of Severe Zoonotic Infectious Diseases, Key Laboratory for Zoonosis Research of the Ministry of Education, Jilin University, Changchun 130062, China; ^2^State Key Laboratory for Molecular Biology of Special Economic Animals, Institute of Special Wild Economic Animals and Plants, Chinese Academy of Agricultural Sciences, Changchun 130112, China; ^3^College of Animal Science and Technology, Yangtze University, Jingzhou 434023, China

**Keywords:** clinical signs, histopathological changes, pathogenicity, porcine circovirus (PCV)

## Abstract

Porcine circoviruses (PCVs) have been a significant concern in swine health, with PCV2 being a well-established pathogen. However, the newly discovered PCV3 and PCV4 have emerged, and their impact on piglets remains less understood. Understanding their pathogenicity is crucial for effective porcine health management. In this study, 3-week-old piglets were inoculated with PCV2, PCV3, and PCV4. PCV2 led to expected growth inhibition and severe clinical signs like anorexia. PCV3, though showing milder symptoms, exhibited unique tissue tropism, with detectable virus in the heart, lungs, and brain tissues. PCV4 caused distinct tissue damage, including cardiac fibrosis and renal changes. In terms of immune responses, each virus triggered different cytokine profiles. PCV3 and PCV4 also demonstrated replication capabilities in vitro and in vivo. PCV3 and PCV4 clearly have pathogenic patterns that differ from those of PCV2. These findings provide essential insights for veterinarians and swine producers. Understanding these viruses' behavior aids in developing targeted prevention and control strategies, such as improved diagnostic tools and potential new vaccines, to enhance porcine health management and safeguard the swine industry from the threats posed by these emerging PCVs.

## 1. Background

Porcine circoviruses (PCVs), which fall within the family *Circoviridae* and the genus *Circovirus*, comprise types ranging from PCV1 to PCV4 [1–3]. PCV1, initially reported in 1974, is commonly regarded as nonpathogenic, as no related diseases have been witnessed in either natural or experimental swine infections [2]. PCV2, discovered in 1998 from pigs afflicted with postweaning multisystemic wasting syndrome (PMWS), has been globally acknowledged in swine herds as the primary pathogen responsible for porcine circovirus-diseases (PCVDs) [4–10]. The most characteristic manifestations of PCV2 include PMWS (also known as PCV2 systemic disease, PCV2-SD), which impacts fattening and growing pigs; porcine respiratory disease complex (PRDC), typically seen in pigs aged 14–20 weeks; PCV2 reproductive disease (PCV2-RD); PCV2 lung disease (PCV2-LD); PCV2 enteric disease (PCV2-ED); PCV2 subclinical infection (PCV2-SI); and acute pulmonary edema. Significantly, PCV2 infection is marked by clinical or subclinical symptoms in swine, resulting in immunosuppression and the failure of vaccines to confer immunity against other pathogens [11–13]. Moreover, the subtypes of PCV2 are expanding, evolving from PCV2a to PCV2h or even further, with PCV2b and PCV2d being the dominant subtypes across the globe [14–16]. Consequently, the prevention and control of PCV infection, particularly the newly identified types or subtypes, pose a formidable challenge.

PCV3 was initially identified in 2016 within US domestic pigs that exhibited clinical signs similar to those of porcine dermatitis and nephropathy syndrome (PDNS), along with cardiac and multisystemic inflammation [1, 17]. Subsequently, PCV3 has been detected globally [18]. PCV3 can infect pigs of all ages, and it has been documented that a correlation exists between PCV3 positivity and the age of the pig, with a higher prevalence of positivity observed in younger pigs [19–21]. Epidemiological studies have reported that the highest PCV3 infection rate was 13.3% among 3–4 week-old suckling piglets, followed by 9.0% in 5–8 week-old weaned piglets, and 9.4% in growers or finishers over 9 weeks old [22].

Furthermore, PCV4 was first identified in 2019 on a pig farm in Hunan, China [3]. Since then, PCV4 has been reported in numerous provinces throughout China, such as Hunan, Henan, Jiangsu, Anhui, Shanxi, Guangxi, and Inner Mongolia [23]. PCV4 has also been detected in Korea, Malaysia, Thailand, Spain, and the USA [24–28]. Hence, the geographical distribution of PCV4 in other regions warrants further exploration.

Previous research findings indicate that PCV4 shares a relatively high nucleotide identity with mink circovirus (66.9%) yet displays lower homology with other PCVs (43.2%–51.5%) [23]. The virus has been detected across all age groups and in aborted fetuses [29, 30]. Surprisingly, no PCV4 has been isolated from field samples thus far. We had previously managed to rescue PCV4 from an infectious clone and discovered that the virus is contagious and pathogenic in piglets [31].

Nevertheless, although PCV3 and PCV4 can be detected in some countries, it remains a matter of debate whether their pathogenicity and potential impact on the pig industry are comparable to those of PCV2. Consequently, it is of utmost urgency to elucidate the pathogenicity and infectivity of PCV3 and PCV4 and to conduct comparisons with the corresponding features of PCV2. In the present study, 3-week-old piglets were inoculated with PCV2, PCV3, and PCV4, respectively, to compare the pathogenicity of these viruses among piglets. The findings of this study are likely to offer a theoretical foundation for the prevention and control of relevant diseases, as well as the development of combined vaccines.

## 2. Materials and Methods

### 2.1. Cells and Viruses

PK-15 cells and the PCV2 strain (GenBank No. JQ955679) were retained in our laboratory. PK-15 cells were cultured with DMEM (Thermo Fisher Scientific, USA) containing 5% FBS (Meilunbio, China) and 1% antibiotics (Mei5Biotechnology, China) in a 39°C, 5% CO_2_ incubator.

The PCV4 was previously rescued from infectious clones in our laboratory [32]. To rescue PCV3, the full-length PCV3 genome (GenBank: MF318451.1) was synthesized and cloned into the pBluescript SK (+) plasmid using restriction sites Hind III and Bam HI, forming the infectious clone pSK-PCV3. Thereafter, PK-15 cells (1 × 10^6^ cells/well) were seeded in a 6-well plate and transfected with 5 µg of pSK-PCV3 plasmid DNA complexed with Lipofectamine 3000 (Thermo Fisher Scientific, USA) in Opti-MEM medium following the manufacturer's protocol. After incubation for 72 h at 37°C in 5% CO_2_, the cells underwent three freeze–thaw cycles to release the virus. The lysate (P0) was inoculated into fresh PK-15 cells for further blind passages (P1, P2, P3, P4, etc.). Virus proliferation and assembly were monitored at each passage using real-time PCR and western blot to confirm the expression of PCV3-specific proteins.

### 2.2. Animal Infection

Specific-pathogen-free (SPF) Danish Landrace piglets (3 weeks old, 5–6 kg each) were purchased from Jilin Agricultural Science and Technology University. Before the experiment, serum from the anterior vena cava of piglets was collected and evaluated for the infections of PCV2, PCV3, PCV4, PRRSV, PRV, CSFV, and PEDV in the piglets using real-time PCR, and negative results for the pathogens mentioned above were obtained. After 1 week of acclimation, piglets were randomly allocated into three experimental groups (*n* = 3): PCV2 group, PCV3 group, PCV4 group, and a control group (*n* = 3) for the viral infection experiment. Each group was housed in a separate room and fed sterile food and water ad libitum. The groups were intranasally inoculated with 5 mL of PCV2, PCV3, and PCV4 (approximately TCID_50_ = 10^4^/mL), respectively. The control group intranasally received an equivalent dose of phosphate-buffered saline (PBS, pH 7.4).

### 2.3. Sample Collection and Processing

The body weight, rectal temperature, and viral load in oral and rectal swabs were documented twice daily before feeding. Clinical manifestations, including but not limited to anorexia, respiratory distress, papules, and diarrhea, were meticulously monitored for any abnormalities. The swab was then placed into a viral transport medium tube, with the piglet's information recorded and stored at −80°C.

At 0, 7, 14, 21, 28, and 35 days postinoculation (dpi), whole blood and serum samples were extracted from the anterior vena cava of piglets. Whole blood was collected into anticoagulant tubes (containing EDTA as an anticoagulant), immediately inverted several times, and stored at 4°C. For serum, whole blood was collected into blood collection tubes (without additives) and centrifuged at 3000 rpm at room temperature for 15 min. The supernatant was transferred to sterile centrifuge tubes and stored at −80°C.

At 35 dpi, piglets were humanely euthanized via intravenous injection of sodium pentobarbital at a dosage of 80 mg/kg body weight, followed by a comprehensive necropsy. Tissues designated for photographic documentation were rinsed twice with PBS buffer before photography. Postphotography, tissues were either snap-frozen in liquid nitrogen or fixed in 4% paraformaldehyde. Tissues collected included the heart, liver, spleen, lungs, kidneys, brain, small intestine, and lymphatic tissues.

### 2.4. Isolation of DNA or RNA

For oral and rectal swabs, before extraction, samples were first homogenized on a vortex mixer before aspirating 200–300 μL of the viral preservation fluid for use. For animal tissues, approximately 20 mg of tissue was placed into a 2-mL grinding tube, to which 1 mL of PBS was added before homogenization using the TGrinder H24R Tissue Homogenizer (Tiangen, Beijing, China), followed by centrifugation at 3000 rpm for 5 min. Then, 200–300 μL of the supernatant was reserved for further use.

Viral genomic DNA from the disrupted tissues, oral, and rectal swab samples were isolated and purified using the Baypure universal magnetic bead method viral DNA/RNA rapid extraction kit and BayBioInvent-K12 automatic nucleic acid extraction system (BayArea Biosciences, Guangzhou, China) according to the manufacturer's instructions. The extracted viral nucleic acids were transferred to a 1.5 mL DNase-free sterile tube and stored at −15 to −25°C or used immediately.

Tissue RNA extraction was performed using the Baypure magnetic bead method total RNA extraction kit and BayBioInvent-K12 automatic nucleic acid extraction system (BayArea Biosciences, Guangzhou, China) according to the manufacturer's instructions. The extracted RNA was transferred to a 1.5 mL sterile, RNase-free tube, stored at −25°C, or used immediately.

### 2.5. DNA or RNA Amplification

Viral genomic DNA was quantified using real-time PCR in a 10 μL reaction system with 2 × SYBR mix (Bimake, China) 5 μL, PCV-qPCR-F 0.5 μL, PCV-qPCR-R 0.5 μL, DNA Template 1 μL, and DNase-free ddH_2_O 3 μL. Primers used in this study are described in Table 1. The real-time PCR parameter was as follows: 95°C 1 min for 1 cycle, 95°C 15 s, 60°C 30 s, and 72°C 30 s for 40 cycles, with the fluorescence threshold set at 0.1 during the exponential phase of amplification. Each sample was tested in triplicate to ensure the reliability of the quantitative real-time PCR results.

The extracted tissue RNA was reverse-transcribed into cDNA to detect cytokine expression levels. The detailed procedure is as follows: 20 μL reaction mixture was prepared containing All-In-One 5 × RT master mix (Bimake, China) 4 μL, RNA Template 1 ng–2 μg, and RNase-Free ddH_2_O to 20 μL. The reverse transcription reaction was conducted under the following conditions: an initial step at 37°C for 15 min, 60°C for 10 min, and 95°C for 3 min. Subsequently, the reaction mixture was cooled on ice and stored at −20°C or used immediately. The real-time PCR protocol was reiterated for subsequent analysis to confirm the consistency and reliability of the results.

### 2.6. Hematoxylin–Eosin Staining (H&E) and Immunohistochemical Staining (IHC)

Tissues were rinsed in PBS, fixed in 4% paraformaldehyde, and then dehydrated in a graded ethanol series from 70% to 100%, each immersion lasting 0.5–1 h. The dehydrated tissues underwent multiple paraffin baths for 12–24 h, were placed in molds, and encased in low melting point paraffin at 4°C. Once solidified, the paraffin-embedded tissues were sectioned (4–8 mm) for H&E Staining or IHC assays.

For H&E staining, paraffin-embedded sections were deparaffinized by heating and then rehydrated through alcohol and water immersions. The sections were stained with 0.5% hematoxylin for 5–10 min, rinsed in alcohol, and stained with 0.5% eosin for 2–5 min. After dehydration and clearing, sections were immersed in xylene, embedded in paraffin, sectioned, mounted on slides, and sealed with a coverslip. The slides were then scanned using the Pannoramic MIDI system (3DHISTECH Ltd., Hungary) and analyzed with CaseViewer software for detailed examination.

For the IHC assay, paraffin-embedded sections were heated at 60°C for 30–60 min, treated with xylene and ethanol gradients, then rinsed in water. Antigen retrieval involved heating sections in 0.01 M sodium citrate buffer (pH 6.0) in a microwave for 4 min, followed by cooling. Sections were blocked with species-matched serum, outlined with an oil-based pen, and treated with primary antibodies specific for PCV2, PCV3, and PCV4, each diluted to 1:100 as previously reported [32], and secondary antibodies. Controls were included to ascertain staining specificity. Then, the sections were stained with DAB-H_2_O_2_ and hematoxylin, differentiated in hydrochloric acid alcohol, and dehydrated. After clearing in xylene, sections were mounted in neutral resin. Scanning was done using Pannoramic MIDI (3DHISTECH Ltd., Hungary) and analyzed with CaseViewer software. Quantitative IHC analysis was performed using ImageJ to measure and calculate the integrated and mean optical density, reflecting protein concentration per unit area. The mean density of selected regions was calculated to determine the sample value.

### 2.7. Enzyme-Linked Immunosorbent Assay (ELISA)

ELISA was employed to test the sera from the experimental and control groups. Briefly, PCV2, PCV3, or PCV4 Cap proteins [32] diluted in coating buffer (2 μg/mL) were added (0.1 mL/well) into a 96-well ELISA plate, followed by overnight incubation at 4°C. After washing with PBST (PBS containing 0.05% Tween-20) three times, the plate was blocked with 0.1 mL of blocking solution (5% FBS in PBST) at 37°C for 2 h. After washing with PBST three times, the plate was added with the sera (1:100 diluted with blocking buffer, 0.1 mL/well) and incubated at 37°C for 1.5 h. Then, the plate was washed with PBST three times, each wash lasting 5 min, and incubated with 0.1 mL of HRP-conjugated goat antipig IgG (H + L) antibody (diluted 1:20,000 in blocking solution, ImmunoWay Biotechnology, USA) at 37°C for 1 h. After washing with PBST three times, 0.1 mL of TMB substrate solution (Solarbio, China) was added into each well and incubated at 37°C in the dark for 10 min. Subsequently, the reaction was stopped by adding 50 μL of 2 M H_2_SO_4_ to each well, and the OD450 nm value was examined using a microplate reader. Each sample should be tested in triplicate.

### 2.8. Virus Isolation

Since PCV2 used in this study was previously isolated from a diseased piglet, while PCV3 and PCV4 were rescued from infectious clones, we tried to isolate PCV3 and PCV4 from the infected piglets to evaluate the feasibility of isolating these two viruses from tissues. Therefore, lungs from PCV3- and PCV4-infected piglets were collected, homogenized, and centrifuged at 3000 × *g* for 10 min. After that, the supernatant was filtered with a 0.22 µm filter and added to 10 cm culture dishes preseeded with PK-15 cells. After 2 h of adsorption at 37°C, the medium was discarded and replaced with DMEM containing 5% FBS and 1% antibiotics. The cells were continuously cultured at 39°C in a 5% CO_2_ incubator for 72 h, after which the virus was harvested using the freeze–thaw method. Then, the virus was blind passaged, and the viral genomic DNA from each passage was identified via real-time PCR.

### 2.9. Transmission Electron Microscope (TEM) Assay

The purified virus particles were negatively stained with 1% phosphotungstic acid and examined using a Hitachi HC-1 transmission electron microscope (Hitachi, Tokyo, Japan) at 80 kV.

### 2.10. Viral Protein Extraction

To prepare viral protein lysates, PCV3- or PCV4-infected PK-15 cells were washed twice with ice-cold phosphate-buffered saline (PBS, pH 7.4) to eliminate residual culture components. Cells were detached by brief digestion with 0.5% trypsin-EDTA (w/v) at 37°C for 1.5 min, then neutralized with a complete DMEM medium containing 10% fetal bovine serum (FBS). The cell suspension was centrifuged at 1000 rpm for 5 min at 4°C to pellet cellular material. After supernatant removal, pellets were resuspended in prechilled western and IP lysis buffer (Beyotime Biotechnology, Shanghai, China) supplemented with 1 mM PMSF (Solarbio, Beijing, China) at a 200 : 1 (v/v) ratio. Homogenized lysates were incubated on ice for 8 min with periodic vortexing to ensure complete membrane disruption. Cellular debris was removed by centrifugation at 10,000 rpm for 15 min at 4°C. The resulting supernatant containing solubilized viral proteins was aliquoted into sterile tubes and stored at −80°C until further analysis.

### 2.11. Western Blot

The western blot was conducted according to the protocol described previously [33]. Total protein lysates were resolved on 12% SDS-polyacrylamide gels (separating gel) with 10% stacking gel under constant voltage (80 V) for 2 h. Separated proteins were electrophoretically transferred onto PVDF membranes at 90 V for 55 min in a 4°C transfer buffer system. Membranes were blocked with 5% (w/v) nonfat dry milk dissolved in Tris-buffered saline containing 0.1% Tween-20 (TBS-T) for 1 h at room temperature with gentle agitation. Following three 10-min washes with TBS-T, membranes were simultaneously probed with both: In-house prepared rabbit polyclonal antibodies against PCV3 or PCV4 capsid proteins (1:2000) [32] and mouse monoclonal β-actin antibody (1:50,000, Proteintech group, China) for 2 h at room temperature to enable parallel detection of viral targets and loading controls. After repeated washing, immunocomplexes were detected using HRP-conjugated goat antirabbit IgG (H + L) secondary antibody (1:10,000, Epizyme, Shanghai, China) with 1.5 h incubation at room temperature. Protein bands were visualized by enhanced chemiluminescence using a super-sensitive ECL luminescence reagent (Meilunbio, Dalian, China) and imaged with a Hesper II imaging system (Monad, Suzhou, China).

### 2.12. Statistical Analysis

Statistical analyses were executed utilizing GraphPad Prism version 5.0 with one-way ANOVA or two-way ANOVA. A *p*-value of less than 0.05 was considered to denote statistical significance. Each experiment was conducted a minimum of three times, and the data are presented as the mean ± standard deviation (SD) of three replicates. *⁣*^*∗*^*p*  < 0.05, *⁣*^*∗∗*^*p*  < 0.01, *⁣*^*∗∗∗*^*p*  < 0.001, *⁣*^*∗∗∗∗*^*p*  < 0.0001.

## 3. Results

### 3.1. Evaluation of PCV2, PCV3, and PCV4 Impact on Clinical Signs in Piglets

**Figure 1:**
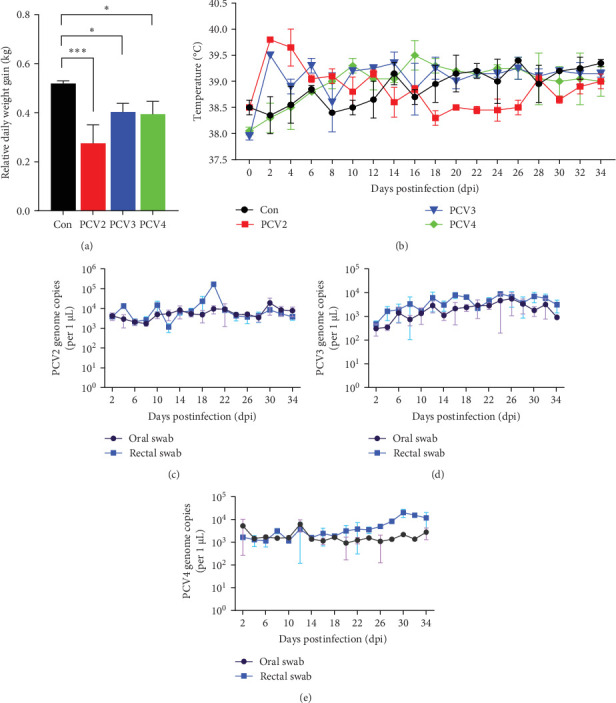
Clinical signs of piglets infected with PCV2, PCV3, and PCV4. Piglets were treated with PBS (Con) or infected with PCV2, PCV3, or PCV4, followed by examining the average daily weight, body temperature, and viral load in oral or rectal swabs at the indicated times. (A) Average daily weight. (B) Body temperature. (C–E) Viral load in oral and rectal swabs from PCV2-infected (C), PCV3-infected (D), and PCV4-infected (E) piglets.

SPF piglets were intranasally administered with PBS or inoculated with PCV2, PCV3, or PCV4, respectively, followed by the documentation of clinical manifestations. As depicted in Figure 1A, during the experimental period, all three infected groups demonstrated varying degrees of growth retardation when compared to the control group (PBS group). The average daily weight gain disparity between the PCV2 group and the negative control was quite pronounced. Meanwhile, significant differences were also noted between the PCV3 or PCV4 groups and the negative control. These findings imply that PCVs, namely PCV2, PCV3, and PCV4, can impede the growth of the infected piglets. Moreover, PCV2 exerted more suppressive effects on the body weight of the infected piglets, followed by PCV3 and PCV4. This result might be because PCV2 was a field isolate, while PCV3 and PCV4 were rescued from infectious clones.

Furthermore, despite the daily fluctuations in body temperature, it remained within the 38–40°C range throughout the experiment, indicating that the piglets' body temperature was normal and no febrile episodes occurred in any of the groups (Figure 1B).

In addition, the clinical manifestations of the infected piglets, such as diarrhea, skin eruptions, respiratory symptoms, loss of appetite, lethargy, and oral and rectal swabs, were monitored. As shown in Table 2, from day 4 onward, diarrhea successively developed in all three experimental groups except for one PCV4-infected piglet. Piglets inoculated with PCV2, PCV3, and PCV4 developed mild skin rashes 4–8 dpi, with lesions in the PCV3 and PCV4 infected groups resolving within approximately 20 days, whereas those induced by PCV2 persisted for a significantly longer duration. All groups of piglets developed mild respiratory symptoms almost simultaneously with skin rashes, primarily characterized by slight wheezing and rapid breathing. Additionally, loss of appetite and lethargy were witnessed in all infected piglets 2 dpi, manifested as diminished appetite, reduced or refusal of feed intake, drowsiness, a preference for lying down, and less standing. These symptoms gradually returned to normal around 14–22 dpi.

Furthermore, oral and rectal swabs were collected every other day after inoculation and analyzed via real-time PCR. As illustrated in Figure 1, in control group piglets, oral and rectal swabs tested negative for PCV2, PCV3, and PCV4, whereas the viral genome was detected in both oral and rectal swabs of piglets inoculated with PCV2, PCV3, or PCV4. The viral load in the oral swabs of the PCV2 group exhibited a relatively stable trend during the experimental period, while the rectal swabs reached the highest copy numbers of 10^5^–10^6^ copies/μL at 20 dpi (Figure 1C). For PCV3, the viral load in both oral and rectal swabs was relatively low during the early stages, followed by a slight increase and stabilization at a plateau level (Figure 1D). Rectal swabs maintained viral copy numbers within the range of 10^3^–10^4^ copies/μL, while oral swab levels were slightly lower than those in rectal swabs. However, the viral copy numbers of PCV3 remained consistently lower compared to PCV2 and PCV4. For PCV4, the viral load in both oral and rectal swabs maintained stable levels at 10^3^–10^4^ copies/μL, with the rectal swabs peaking around 10^4^–10^5^ copies/μL at 30 dpi (Figure 1E). These results suggest that PCV2 and PCV4 can replicate more effectively in the piglets than PCV3, resulting in relatively higher virus loads in both oral and rectal swabs. At the same time, PCV3 exhibited relatively lower replication efficiency and viral shedding levels.

### 3.2. Differential Pathogenic Effects of PCV2, PCV3, and PCV4 on Piglet Organs at 35 dpi

**Figure 2:**
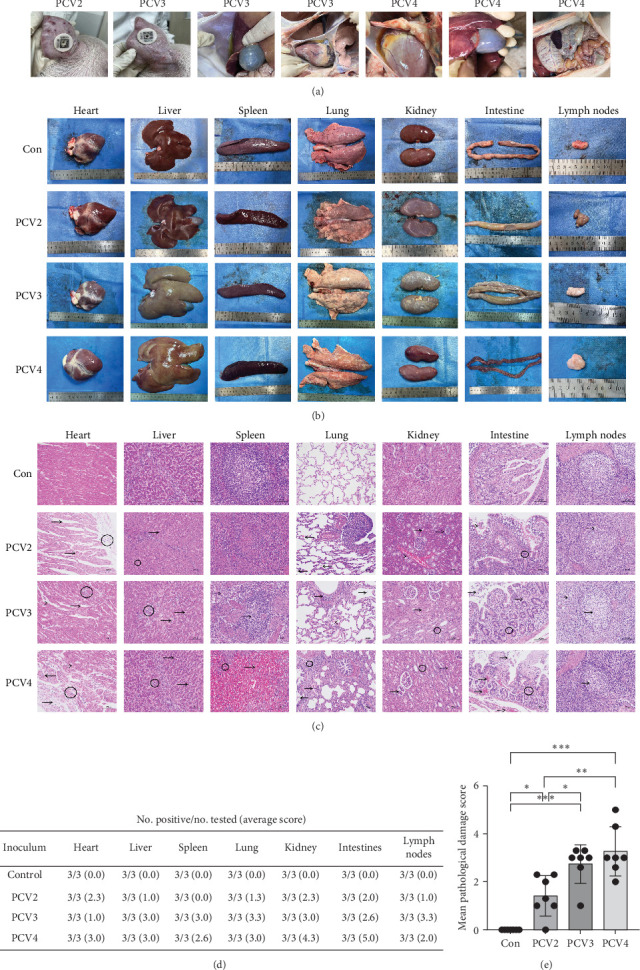
Gross lesion and histopathological analysis in PCV-infected piglets. (A) Necropsy photographs illustrating gross pathology in piglets infected with PCV2, PCV3, and PCV4, compared to negative control (Con). (B) Organ comparison between control and infected groups, displaying visible pathological changes in the heart, liver, spleen, lungs, kidneys, intestines, and lymph nodes. (C) Histological sections stained with hematoxylin and eosin (H&E) show tissue architecture and the degree of inflammatory infiltration (circle) or damage (arrows) in the corresponding organs of Control, PCV2, PCV3, and PCV4 infected piglets. (D and E) Histopathological scores of tissues in the control and PCV-infected piglets. Average histological scores for tissues: 0, No lesions (absence of pathological changes); 1, Minimal (slight pathological changes, focal and limited); 2, Mild (localized pathological changes with mild tissue involvement); 3, Moderate (distinct pathological changes affecting ≤30% of the tissue); 4, Severe (extensive pathological changes affecting >30% of the tissue); 5, Profound (extreme tissue damage with structural disruption). *⁣*^*∗*^*p* < 0.05, *⁣*^*∗∗*^*p* < 0.01, *⁣*^*∗∗∗*^*p* < 0.001.

To explore the differences in PCV2, PCV3, and PCV4 pathogenicity within piglets, organs such as the heart, liver, spleen, lungs, kidneys, intestines, and lymph nodes were collected and examined at 35 dpi. As anticipated, no notable pathological alterations were witnessed in the control group (Figure 2). In contrast, pathological changes were detected in the organs of pigs inoculated with PCV2, PCV3, and PCV4. Hemorrhages or dermatitis in the ears were noticed in piglets infected with PCV2 and PCV3 (Figure 2A). Moreover, piglets infected with PCV3 and PCV4 displayed pleural and pericardial effusion. Piglets infected with PCV4 presented extensive multiorgan hemorrhaging.

As depicted in Figure 2B, the apices of the hearts in the PCV2 group were relatively pointed. The hearts in the PCV3 group had an irregular shape. The livers of the PCV2 group were darker in hue. The livers in the PCV3 group exhibited uneven coloration and a coarse texture. The livers of both the PCV3 and PCV4 groups showed signs of jaundice. The spleens in the PCV2 and PCV4 groups were darker than those in the control, accompanied by indications of congestion or hemorrhage. The lungs of the PCV2 group had uneven color changes on the surface, along with petechial hemorrhages. The lungs of the PCV3 and PCV4 groups were characterized by fibrosis, with pale white patches and fibrous strands. The kidneys of the PCV2 group had several scattered white spots on the surface. The kidneys of the PCV3 group had numerous gray-white necrotic foci. The kidneys of the PCV4 group demonstrated hemorrhage. The intestinal surfaces of the PCV2 and PCV3 groups presented transparent or semi-transparent vacuoles. In contrast, the intestines of the PCV4 group were dark purple overall, with evident bleeding. The lymph nodes in the PCV-infected groups did not show significant differences from the control. The observed variations underscore the differential pathogenicity of PCVs towards various organs.

The results of H&E staining revealed that piglets infected with PCV2, PCV3, and PCV4 exhibited significant histopathological changes, highlighting the distinct pathogenic impacts of each virus (Figure 2C). In the heart, infections with PCV2, PCV3, and PCV4 led to varying degrees of myocardial fiber edema, widening of the interstitial spaces in the endocardium and interstitium, as well as inflammatory cell infiltration. Among them, the myocardial cell damage in the PCV4 infection group was the most severe.

In the liver, PCV2 infection resulted in slight focal necrosis of hepatocytes, accompanied by mild nuclear dissolution, dilation of the hepatic sinusoids, and inflammatory cell infiltration. Infection with PCV3 caused focal necrosis of hepatocytes around the central veins, characterized by nuclear pyknosis and fragmentation, inflammatory cell infiltration, and slight fatty degeneration of hepatocytes. Infection with PCV4 led to moderate hepatocyte edema and mild fatty degeneration of hepatocytes. In the portal areas, there was edema of the bile duct epithelial cells and mild inflammatory cell infiltration. Among them, the PCV4 infection group exhibited mild bile duct damage.

The white pulp cells in the PCV3 infection group were loosely arranged in the spleen, and the number of red pulp cells in the spleen was significantly reduced. The PCV4 infection group displayed extensive hemorrhage in the red pulp, with an indistinct white pulp structure and a dispersed distribution of lymphocytes.

In the lungs, infections with PCV2, PCV3, and PCV4 led to degeneration of the epithelial cells of the bronchioles. Furthermore, in the PCV4 infection group, there was apparent interstitial pneumonia, with perivascular exudation of inflammatory cells, and the alveolar walls showed inflammatory cell infiltration and thickening.

In the kidneys, the PCV2 group exhibited glomerular atrophy, interstitial vasodilation, and slight fatty degeneration of the epithelial cells in the proximal convoluted tubules. Both PCV3 and PCV4 had vacuolar degeneration of glomerular epithelial cells, and inflammatory cells were diffusely distributed in the interstitium. PCV4 is also associated with glomerular atrophy.

In the intestines, the PCV2 group showed a disordered structure of intestinal villi, with partial mucosal epithelial shedding, moderate inflammatory cell infiltration in the lamina propria, irregular arrangement of intestinal glands, and edema of glandular cells. PCV3 infection led to extensive shedding of mucosal epithelium, moderate inflammatory cell infiltration in the lamina propria, and loose arrangement of intestinal glands. PCV4 intestinal villi dissolve and break, intestinal villi fragments in the intestinal lumen, and diffuse distribution of inflammatory cells in the interstitium

There is cellular vacuolation and a small amount of cellular debris in the lymphoid and cortical paracortex of the PCV2 group. In the PCV3 and PCV4 infection groups, cellular vacuolation in the lymphatic paracortical nodules and regions reduced the number of lymphocytes in germinal centers. These results indicate significant alterations in the lymphatic system across different PCV infections.

Furthermore, histopathological lesions in the heart, liver, spleen, lung, kidney, lymph nodes, and intestines were scored according to a previously published scoring system [34]. The mean lesion scores of tissues in PCV2-, PCV3-, and PCV4-inoculated piglets showed significant differences from those in control group piglets (*p*  < 0.05; Figure 2D,E). Notably, the mean pathological damage scores of these tissues in PCV3- and PCV4-inoculated groups were higher than those in the PCV2-inoculated group, whereas no significant difference was observed between PCV3- and PCV4-inoculated groups.

Based on the detailed histopathological examination of piglets infected with PCV2, PCV3, and PCV4, it can be concluded that each virus induces distinct and significant modifications across various organs. These changes encompass myocardial damage, disruption of liver structure, damage to the white pulp and red pulp in the spleen, inflammatory changes in the bronchioles and interstitium of the lungs, glomerular atrophy, and severe gastrointestinal damage. Additionally, inflammatory cell infiltration across most organs emphasized the inflammatory response triggered by the infections. These findings demonstrate that PCV2, PCV3, and PCV4 can cause extensive and diverse histopathological changes in piglets.

### 3.3. The Distribution Profile of PCV2, PCV3, and PCV4 in Porcine Tissues

**Figure 3:**
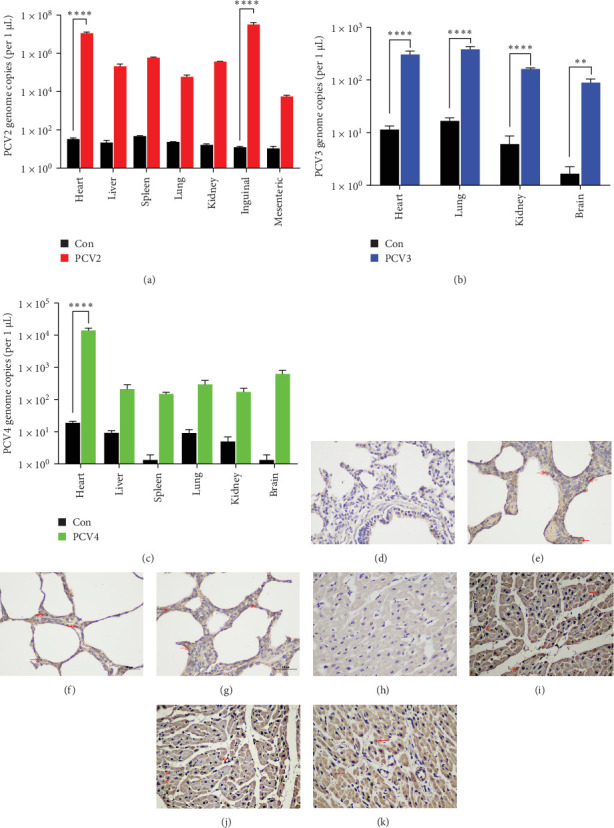
Viral distribution and immunohistochemical analysis in tissues of PCV-infected piglets. (A–C) Bar graphs showing the quantification of PCV2, PCV3, and PCV4 genomic copies in different piglet tissues. (D–K) IHC staining for PCV antigens in heart and lung tissues. Antigen-positive cells are indicated by brown staining. (D) No positive cells were observed in lung sections from control piglets. (E–G) Lung tissues from PCV2- (E), PCV3- (F), and PCV4-inoculated (G) piglets exhibited numerous antigen-positive cells (arrows). (H) No positive cells were detected in heart sections from control piglets. (I–K) Heart tissues from PCV2- (I), PCV3- (J), and PCV4-inoculated (K) piglets showed abundant antigen-positive cells (arrows). Con, control.

To assess the viral genomic DNA, real-time PCR was employed to evaluate PCV2, PCV3, and PCV4 distribution patterns within piglets. The results revealed that varying amounts of viral genomic DNA were detected in the diverse tissues of the piglets infected with PCVs, while no positive results were obtained in the control group. This result demonstrated the broad tissue tropism of PCV2, PCV3, and PCV4 in piglets (Figure 3). Moreover, PCV2 was detectable in nearly all tissues except for the brain (Figure 3A). PCV3 was verified in the heart, lungs, kidneys, and brain of the infected piglets (Figure 3B). For PCV4, its viral genomic DNA could be identified in the heart, liver, spleen, lungs, kidneys, and brain, suggesting wide-spectrum infectivity (Figure 3C). Notably, in contrast to PCV2, viral DNA was detected in the brains of piglets infected with both PCV3 and PCV4, indicating the potential of PCV3 and PCV4 to trigger neurological disorders in the infected piglets.

Additionally, immunohistochemical analyses were performed to detect the presence of PCV antigens in each group's heart and lung tissues. As shown in Figure 3, samples from control piglets tested negative for PCV antigens in both heart and lung tissues. However, tissues from piglets infected with PCV2, PCV3, or PCV4 exhibited positive signals for specific antibodies against PCV2, PCV3, or PCV4 (anti-PCV2, anti-PCV3, or anti-PCV4 antibodies, respectively) in cardiomyocytes and alveolar epithelial cells.

### 3.4. Profiling of Immune Responses Induced by PCV2, PCV3, and PCV4 in Piglets

**Figure 4:**
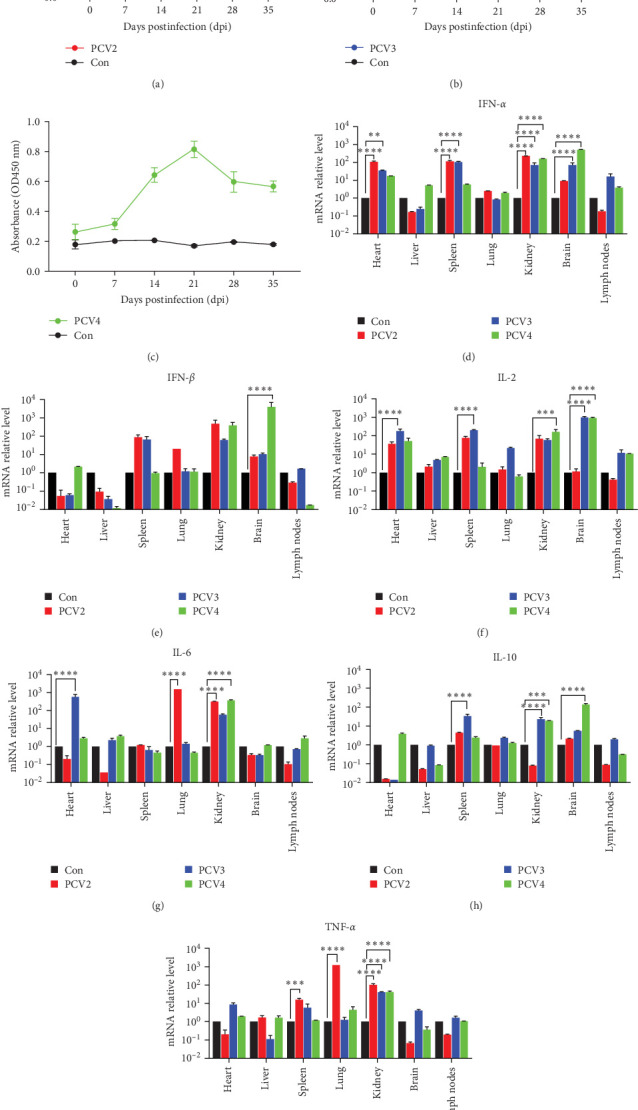
Cytokine response in PCV-infected piglets. (A–C) Line graphs represent ELISA-based antibody responses over time, illustrating the immune response dynamics throughout the infection for PCV2 (A), PCV3 (B), and PCV4 (C). (D–I) Bar graphs display the relative mRNA levels of immune markers in piglet tissues infected with PCV2, PCV3, and PCV4 compared with the control piglets. (D) IFN-α, (E) IFN-β, (F) IL-2, (G) IL-6, (H) IL-10, and (I) TNF-α. The graphs demonstrate significant upregulation of these cytokines in infected tissues, indicative of an immune response to viral infection, with asterisks denoting statistical significance compared to the control (Con).

To explore the antiserum levels in piglets infected with PCVs, ELISA was employed to measure PCV-specific antibody levels at 0, 7, 14, 21, 28, and 35 dpi. The results showed that PCV-specific antibodies increased significantly, reaching a peak at either 21 or 28 dpi in the infected piglets, and then gradually declined until the end of the experiment (Figure 4A–C). In contrast, no substantial alterations in PCV-specific antibodies were detected in the control group. The observed antibody dynamics imply that piglets exposed to PCVs initiated an effective humoral immune response, resulting in changes in serum antibody levels.

Furthermore, real-time PCR was used to assess the levels of cytokines, including IFN-α, IFN-β, IL-2, IL-6, IL-10, and TNF-α in various tissues of piglets inoculated with PCVs. The findings indicated that IFN-α levels were markedly elevated in the kidneys of all the PCV-infected groups (Figure 4D). A significant upregulation of IFN-α expression was also noticed in the heart and spleen for the PCV2 and PCV3 groups, and an increase in IFN-*α* was observed in the brains of the PCV3- and PCV4-infected piglets. Additionally, PCV4 significantly boosted IFN-β levels in the brain (Figure 4E), while no significant difference was detected in other groups. IL-2 was highly expressed in the heart and spleen of the PCV3 group and the kidney of the PCV4 group, and both PCV3 and PCV4 significantly raised IL-2 levels in the brain (Figure 4F). IL-6 was induced in the heart of the PCV3 group, the lung of the PCV2 group, and the kidney of the PCV2- and PCV4 groups (Figure 4G). A significant enhancement of IL-10 was detected in the spleen of the PCV3 group, the kidneys of the PCV3- and PCV4 groups, and the brain of the PCV4 group (Figure 4H). For TNF-α, a significant increase was observed in the spleen and lung of the PCV2-infected group and the kidneys of the three infected groups (Figure 4I). Notably, significant decreases were detected in the heart and lymph nodes of PCV2-infected piglets.

These results emphasize the intricate interplay of immune responses triggered by PCVs in piglets. The variations in cytokine expression across different organs signify a subtle and nuanced immune reaction to these viral challenges, which may offer valuable insights into the pathogenesis and potential immune targets for intervention.

### 3.5. Isolation and Cultivation of PCV3 and PCV4 From Lung Tissues

**Figure 5:**
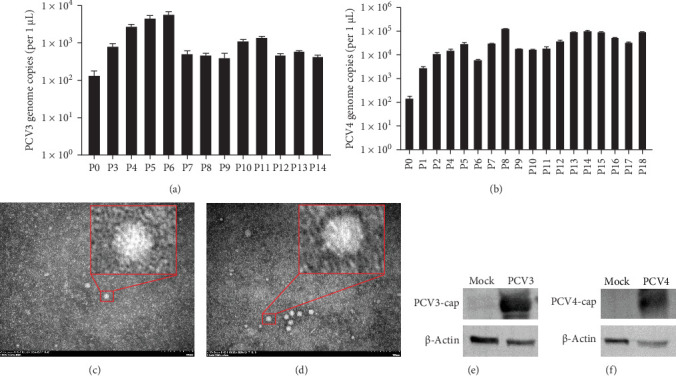
Quantification of PCV3 and PCV4 viral loads in serial passages. (A) Bar chart depicting the number of PCV3 (A) or PCV4 (B) genomic copies over successive passages. P, passage. (C and D) Transmission electron micrographs of purified PCV3 (C) and PCV4 (D) isolates at different passages. Scale bars = 200 nm. (E and F) Western blot validation of PCV3 (E, ~25 kDa) and PCV4 (F, ~27 kDa) capsid proteins in purified isolates of passage 10. Mock: uninfected PK-15 cell.

Given that the PCV3 and PCV4 utilized in this study were rescued from infectious clones, we assessed whether PCV3 and PCV4 could be isolated from the infected piglets and propagate in vitro. Consequently, lungs were collected and homogenized, and the resulting homogenates were then incubated in PK-15 cells for subsequent passages. As depicted in Figure 5A,B, both PCV3 and PCV4 were successfully isolated from the lungs of the infected piglets. Moreover, the isolated viruses could be stably passaged within PK-15 cells. The highest copy numbers reached approximately 6.8 × 10^3^ for PCV3 and 1.2 × 10^5^ for PCV4, respectively. The isolated virus was observed under the electron microscope, and the virus particles of about 20 nm could be observed, which proved that the virus was isolated from the lungs (Figure 5C,D). Viral proteins were detected via western blot, and specific bands of the expected size were obtained, ranging from approximately 25 kDa for PCV3 and 27 kDa for PCV4, and not in the control uninfected PK-15 cells (Figure 5E,F). Besides, Comparative analysis with the original infectious clone sequences (GenBank: PCV3_MF318451.1, PCV4 MT311854.1) showed 99.96% (PCV3 P3, P7, and P10) or 99.94% (PCV4 P5, P10, and P15) genomic sequence identity, with 100% nucleotide conservation at key replication-associated motifs (Figures S1 and S2).

These findings further highlight that the rescued viruses, PCV3 and PCV4, were replicable, infectious, and pathogenic in vivo. It is worth noting that PCV4 exhibited more efficient replication than PCV3 in vitro. To our knowledge, this is the first report describing the successful isolation and stable in vitro propagation of PCV4 from experimentally infected piglets, which provides a valuable resource for future studies on PCV4 biology and pathogenesis.

## 4. Discussion

PCV2, renowned for its pathogenic nature, has drawn global attention since 1998. It is the principal pathogen in PCVD [7, 10]. In this study, piglets infected with PCV2 demonstrated growth retardation and clinical manifestations like loss of appetite and lethargy. Pathological alterations were observed as well. The darker appearance of the livers could be ascribed to moderate blood stasis, hinting at systemic circulatory disruptions or inflammatory reactions. The spleens exhibited enhanced filtering functions and moderate congestion, corresponding to an active immune response. In the kidneys, the white spots were more conspicuous, potentially signifying localized fibrosis or mild inflammation. On the lung surfaces, variable pigmentation along with several hemorrhagic areas were present, probably indicating moderate acute lesions or congestion, in line with the pulmonary stress triggered by PCV2. Tissue sections disclosed damage to the lymphatic tissue, such as the disappearance of B-cell follicles in lymph nodes, which signified a weakened immune system. Moreover, blood stasis or mild inflammation in the liver and kidneys reflected localized damage. These pathological changes were indicative of PCV2-SD, which is characterized by emaciation, respiratory distress, lymphadenopathy, diarrhea, pallor, and jaundice [35, 36]. Histopathological lesions encompassed the loss of B-cell follicles in lymph nodes, lymphocyte-histiocyte infiltration in the lungs, liver, and kidneys, depletion of mature lymphocytes in the spleen, and atrophy of pancreatic acinar cells [36].

Since the initial report of PCV3, it has been extensively detected in organs and tissues such as the lungs and heart [37], giving rise to a diverse range of clinical manifestations [20]. Current research reveals multiple confounding elements influence the clinical presentation and pathological alterations in affected pigs [20, 21]. Coinfections of PCV3 with other viruses, including PCV2, CSFV, PRRSV, PPV, and TTSuV, have been documented [18, 38]. Hence, detecting PCV3 in samples does not always correspond to disease mortality [39]. Recently, a study also emphasized the potential role of PCV3 in subclinical infections, which are characterized by the absence of apparent clinical signs yet the presence of detectable prolonged viremia, viral replication in tissues, and multisystemic inflammation [40]. In this study, piglets infected with PCV3 displayed significant symptoms like weight reduction, breathing difficulties, and diarrhea, which implied systemic inflammatory responses. Pathological changes witnessed in PCV3-infected piglets encompassed slight surface irregularities on the heart, mild uneven coloration and texture of the liver, and faint gray–white discolorations in the kidneys, all signified localized renal stress. The lungs presented pale white patches and mild fibrous streaks, suggesting moderate pulmonary modifications. These observations demonstrated cardiac, hepatic, renal, and pulmonary responses to PCV3 infection. However, these responses seemed less severe and more localized than those induced by other PCVs, indicating a unique pathogenic influence of PCV3. Tissue sections further disclosed myocardial and hepatic cell damage, as well as chronic inflammation, along with slight damage in the kidneys and lungs, reflecting the chronic lung disease and multiorgan involvement caused by PCV3. Notably, the replication ability of PCV3, both in vivo and in vitro, was weaker than that of PCV2 and PCV4, as evidenced by the low copy numbers in the swab (Figure 1) and infected cells (Figure 5). Nevertheless, the outcome of tissue pathological changes was as severe as that of the other two viruses (Figure 2), suggesting that PCV3 might possess relatively higher pathogenicity than the other two despite its lower replication capacity. Furthermore, the results suggest that the in vivo replication ability of PCV3 is weaker than that of PCV2 and PCV4. This results in a lower viral load within infected pigs. Moreover, PCV3 exhibits distinct tissue tropism, favoring specific tissues like the heart, lungs, kidneys, and brain. In contrast to PCV2 and PCV4, PCV3 may rely more on bloodborne or tissue-specific pathways for transmission rather than being secreted through oral and rectal routes. Besides, PCV3 might adopt a more nuanced infection strategy, triggering less pronounced inflammatory responses or following a different viral dissemination pattern, thus restricting the degree to which the virus is shed into oral and rectal secretions. To sum up, the low copy numbers of PCV3 in oral and rectal swabs are likely due to its weaker replication ability, distinct tissue tropism, and unique transmission mechanisms rather than signifying a lack of infectivity or pathogenicity compared to other PCVs. However, the exact mechanism needs to be clarified in future research.

Up to now, only a limited amount of research has centered on the pathogenicity of PCV4, a newly discovered porcine circovirus. PCV4 can be detected in piglets with signs of respiratory and enteric diseases, PDNS, and PMWS [30, 41]. The clinical manifestations associated with PCV4 infection encompass diarrhea, pulmonary edema, skin lesions, neurological symptoms, miscarriages, and stillbirths. Pigs displaying clinical signs of PCV4 infection have also been found to be coinfected with other PCVs, such as PCV2 and PCV3 [30]. Like PCV3, PCV4 has been identified in clinically healthy pigs and pigs coinfected with PCV2 and PCV3 [26, 41]. Although PCV4 was first reported in 2019, retrospective studies have demonstrated that it has been prevalent in China since 2012 [42]. In the current study, piglets infected with PCV4 exhibited signs and lesions comparable to those seen in piglets infected with PCV2 and PCV3, including weight reduction, breathing difficulties, and diarrhea, which reflects the extensive impact of PCV4 on the overall health of piglets. Symptoms like loss of appetite and fever indicated systemic inflammation triggered by PCV4. Pathologically, PCV4 induced moderate alterations, such as cardiac fibrosis, mild bile duct damage, and localized splenic hemorrhage. The kidneys and lungs manifested acute changes and fibrosis, suggesting structural damage and inflammation within these organs. These findings imply chronic tissue stress, potentially similar to the mechanisms underlying chronic obstructive pulmonary disease. While certain results in this study deviated from our previous research on PCV4-infected piglets [31], for instance, the absence of clinical symptoms like rashes and variations in histopathological changes observed in earlier studies, these differences might be ascribed to disparities in the age of the piglets used. In this study, 3-week-old piglets, younger than those in the previous experiments (5-week-old piglets), were employed, and the intranasal inoculation dosage also varied. Nevertheless, PCV4 can replicate in vivo and in vitro across a relatively wide range of piglet ages, inducing pathological changes of different severities. Given the similarities in pathogenicity among PCV4, PCV2, and PCV3, it can be hypothesized that PCV4 may also become endemic on a global scale.

Importantly, this study represents the first successful isolation of PCV4 from experimentally infected piglets. The virus was isolated from lung tissues and propagated in PK-15 cells over multiple passages, confirming its replication competence in vitro. The ability to isolate and culture PCV4 validates its pathogenic potential observed in vivo and provides a valuable resource for future studies to elucidate PCV4's replication dynamics, host interactions, and the development of targeted vaccines or therapeutic strategies. This achievement marks a significant step forward in PCV4 research, offering new opportunities to investigate the molecular mechanisms underlying PCV4-associated diseases.

Evidence indicates that PCVs predominantly infect monocytes, enabling them to subsequently infect various cell types, such as monocytes, macrophages, dendritic cell (DC) precursors, myeloid DCs, and plasmacytoid DCs [43–45]. These cells do not serve as the principal sites for viral replication but rather facilitate the spread of the virus to diverse tissues [46]. In this study, PCV2 DNA was detected across nearly all the tested tissues, demonstrating that PCV2 has a broad distribution within these tissues and possesses a robust replication ability. This tissue tropism might be crucial in the multisystemic diseases resulting from PCV2 infection. Moreover, PCV3 DNA was detected in the heart, lungs, kidneys, and brain tissues. The detection in brain tissues suggests a potential connection to neurological diseases, which aligns with the reports by Phan et al. [17], emphasizing the association of PCV3 with neurological disorders. This result also corroborates previous studies indicating that PCV3 may trigger multisystemic diseases, including those affecting the nervous system. The situation regarding PCV4 is analogous to that of PCV2 and PCV3. IHC results have confirmed the extensive distribution of PCV4 in multiple tissues, including the heart, liver, spleen, lungs, kidneys, and brain. Identifying the virus in brain tissues is especially significant, hinting that PCV4 may also impact the nervous system. These findings disclose the wide tissue distribution of PCV2, PCV3, and PCV4, underlining their potential to induce related diseases, particularly their possible influence on the nervous system. Future research should focus on exploring the pathogenic mechanisms of these viruses and their replication dynamics within the host to more effectively prevent and control the diseases caused by these viruses.

Furthermore, the influence of PCV2, PCV3, and PCV4 on the immune response in piglets was assessed by evaluating the expression levels of cytokines in various tissues. Examining immune factors in infected piglets disclosed a significant upregulation of cytokines, including IFN-α, IFN-β, and IL-2 in the heart, spleen, and kidneys. Of note was the substantial increase in IFN-*α* within the brain tissues of the PCV3 and PCV4 groups, which might be associated with neurological lesions. Remarkably, significant decreases were detected in the heart and lymph nodes of PCV2-infected piglets. This result could potentially account for the well-documented immunosuppression caused by PCV2 in practical scenarios, which has been attributed to lymphoid tissue depletion, apoptosis of immune cells, and cytokine dysregulation [47]. This meticulous analysis of cytokine expression underlines the intricate immune responses elicited by PCV infections, stressing the significance of comprehending these mechanisms for more effective management and control of PCV-related diseases.

Moreover, starting from 7 dpi, notable increases in PCV-specific antibody levels were witnessed, suggesting that the infected piglets could initiate an effective humoral immune response. Notably, while PCV2 infection resulted in both cytokine dysregulation and lymphoid suppression, PCV3 and PCV4 infections were associated with heightened cytokine responses and consistent antibody production. These findings suggest that unlike PCV2, PCV3, and PCV4 may not induce overt immunosuppression but instead trigger complex immune modulation, characterized by simultaneous activation of antiviral cytokines and potential regulatory signals such as IL-10. However, further investigations are required to fully elucidate the long-term immunological consequences of PCV3 and PCV4 infections and to determine whether the observed immune activation might lead to immune exhaustion or dysregulation over time.

## 5. Conclusions

In summary, PCV2, PCV3, and PCV4 display pathogenic properties in piglets, demonstrating similarities and distinctions in clinical manifestations, tissue lesions, and viral distribution. The outcomes of this study offer vital information that facilitates a more profound comprehension of the pathogenic mechanisms of these viruses, enables an evaluation of their potential menace to the swine industry, and supports the development of effective prevention and control strategies. Future research endeavors should clarify the pathogenic mechanisms of these viruses, particularly how they impact the nervous system of piglets and the possible interactions among them.

## Figures and Tables

**Table 1:** Primers used in this study.

Primer name	Sequence (5′–3′)	Length (bp)
Pig IFN-α-F	5-GGCTCTGGTGCATGAGATGT-3	336
Pig IFN-α-R	5-GCCTTCTTCCTGAATCTGTCTTA-3	
Pig IL-2-F	5-CTCTCCAGGATGCTCACATTTA-3	96
Pig IL-2-R	5-CTCCAGAGCTTTGAGTTCTTCT-3	
Pig IL-6-F	5-ATCCAGACCCTGAGGCAAAA-3	200
Pig IL-6-R	5-AGGTGCCCCAGCTACATTAT-3	
Pig TNFα-F	5-CCTACTGCACTTCGAGGTTATC-3	117
Pig TNFα-R	5-ACGGGCTTATCTGAGGTTTG-3	
Pig IL-10-F	5-TGTAATGCCGAAGGCAGAGA-3	171
Pig IL-10-R	5-TGGAGCTTGCTAAAGGCACT-3	
Pig IFN-β-F	5-CCTCCAAATCGCTCTCCTGA-3	129
Pig IFN-β-R	5-GGAGTCCCAGGCAACTGTTC-3	
Pig GAPDH-F	5-GCCATCACCATCTTCCAGG-3	190
Pig GAPDH-R	5-TCACGCCCATCACAAACAT-3	
PCV2-qPCR-F	5-TGTAGTATTCAAAGGGCACAGAGC-3	131
PCV2-qPCR-R	5-CGGATATACTATCAAGAAAACCAC-3	
PCV3-qPCR-F	5-AGAGGCTTTGTCCTGGGTGAG-3	131
PCV3-qPCR-R	5-GCTCCAAGACGACCCTTATGC-3	
PCV4-qPCR-F	5-GCAAGTGGTGGGATGGATATAA-3	96
PCV4-qPCR-R	5-TGTCCATGAGTCTCAACAAGTC-3	

**Table 2:** Clinical sign score of piglets.

	Diarrhea			Skin rashes			Respiratory signs			Loss of appetite			Reduced activity		
	Max score	Appeared time	Duration of onset	Max score	Appeared time	Duration of onset	Max score	Appeared time	Duration of onset	Max score	Appeared time	Duration of onset	Max score	Appeared time	Duration of onset
Control	0	/	/	0	/	/	0	/	/	0	/	/	0	/	/
PCV2	2–4	Day 4	32 days	3	Days 4–8	27–32 days	1	Day 4	12–16 days	4	Day 2	12–14 days	5	Day 2	12–14 days
PCV3	2–3	Day 6	30 days	1–2	Day 6	18–20 days	1–2	Days 4–6	14–20 days	3–4	Day 2	14–20 days	4	Day 2	20–22 days
PCV4	2–3	Day 4	14–32 days	2–3	Days 4–8	18–20 days	2–3	Days 4–8	30–32 days	4	Day 2	20–22 days	4	Day 2	20–22 days

*Note:* Sign severity scoring scale: Clinical signs are scored on a scale from 0 to 5, with 0 indicating no signs, 1 for very mild signs, 2 for mild signs, 3 for moderate signs, 4 for severe signs, and 5 for more severe signs.

## Data Availability

All data generated or analyzed during this study are included in this published article.

## Supporting Information

Supporting Information Figures S1 and S2. Comparative analysis with the original infectious clone sequences (GenBank: PCV3_MF318451.1, and PCV4 MT311854.1) showed 99.96% (PCV3 P3, P7, and P10) or 99.94% (PCV4 P5, P10, and P15) genomic sequence identity, with 100% nucleotide conservation at key replication-associated motifs.
