# Supplementary material for: Comparative Pathogenicity of PCV2, PCV3, and PCV4 in Piglets: Insights Into Clinical, Pathological, and Immunological Features
Source: Transbound Emerg Dis. 2025 Jul 9;2025:6362100. doi: 10.1155/tbed/6362100 (PMC12267972; doi:10.1155/tbed/6362100)
Supplement: Supporting Information — Figures S1 and S2. Comparative analysis with the original infectious clone sequences (GenBank: PCV3_MF318451.1, and PCV4 MT311854.1) showed 99.96% (PCV3 P3, P7, and P10) or 99.94% (PCV4 P5, P10, and P15) genomic sequence identity, with 100% nucleotide conservation at key replication-associated motifs. [file 6362100.f1.docx]

Supplemental file for

Comparative Pathogenicity of PCV2, PCV3, and PCV4 in Piglets: Insights into Clinical, Pathological, and Immunological Features

Jiawei Zheng ^a, †^, Xue Li ^a, †^, Xinru Lv ^a^, Yaqi Han ^a^, Xinwei Zhang ^a^, Si Chen ^b^, Fuxian Zhang ^c^ and Linzhu Ren ^a, c, #^

**Supplemental Fig S1 to S2**

**Supplemental Figures**

**Fig. S1** Comparative analysis with the original infectious clone sequence (GenBank: PCV3_MF318451.1) showed 99.96% (PCV3 P3, P7, P10) genomic sequence identity, with 100% nucleotide conservation at key replication-associated motifs.

**Fig. S2** Comparative analysis with the original infectious clone sequence (GenBank: PCV4 MT311854.1_) showed 99.94% (PCV4 P5, P10, P15) genomic sequence identity, with 100% nucleotide conservation at key replication-associated motifs.
